# Survey of Phytochemical Composition and Biological Effects of Three Extracts from a Wild Plant (*Cotoneaster nummularia* Fisch. et Mey.): A Potential Source for Functional Food Ingredients and Drug Formulations

**DOI:** 10.1371/journal.pone.0113527

**Published:** 2014-11-19

**Authors:** Gokhan Zengin, Ahmet Uysal, Erdogan Gunes, Abdurrahman Aktumsek

**Affiliations:** 1 Selcuk University, Science Faculty, Department of Biology, Konya, Turkey; 2 Selcuk University, Vocational School of Health Services, Konya, Turkey; National Taiwan University, Taiwan

## Abstract

This study was focused on the analysis of the phenolic content, antioxidant, antibacterial, anti-cholinesterase, anti-tyrosinase, anti-amylase and anti-glucosidase activity of three solvent extracts from *Cotoneaster nummularia*. Moreover, water extract was tested in terms of mutagenic/anti-mutagenic effects. The antioxidant activities of these extracts were evaluated by DPPH, ABTS, O_2_, metal chelating, phosphomolybdenum, β-carotene/linoleic acid, ferric and cupric reducing power assays. Enzyme inhibitory activities were also examined with colorimetric methods. Generally, methanol and water extracts exhibited excellent biological activities. These extracts were rich in phenolic and flavonoid content. Furthermore, *Cotoneaster* extracts indicated appreciable antibacterial properties against human pathogen strains. HPLC analysis showed that ferulic acid, chlorogenic acid, (-) – epicatechin and (+)-catechin were the major phenolics in extracts tested. These data offer that these extracts from *C. nummularia* may be considered as a potential source of biological agents for developing functional foods or drug formulations.

## Introduction

In the past decade, an increasing interest in the use of natural bioactive compounds origin from plant for scientific research as well as different purposes such as pharmaceutical and food industries [1]. For example, plant antioxidants are very significant for aforementioned areas, because many synthetic antioxidants (butylated hydroxyanisole (BHA), butylated hydroxyltoluene (BHT) and propyl gallate (PG)) have possible activity as promoters of carcinogenesis [2]. In addition, natural products are an important group of preventive agents for the treatment of global health problems such as Alzheimer's diseases (AD), diabetes mellitus (DM). Inhibition of the key enzymes became a widely used treatment strategy in the pathogenesis of AD and DM. To this end, many enzyme inhibitors are produced synthetically for these diseases. For instance, Galatamine, tacrine are employed to be cholinesterase inhibitors to treat AD. Likewise, acarbose and viglibose are accepted as powerful inhibitors on α-amylase and α-glusosidase for DM. On the other hand, several reports have revealed that the synthetic enzyme inhibitors have certain adverse effects including liver damage, gastrointestinal disturbances [3]–[6]. From this point of view, many efforts are performed to search for more effective and safe inhibitors of key enzymes from plants to develop natural agents to treat these diseases.

Human environment consistently encounters with mutagenic and carcinogenic agents and eradication of these agents appear to be very laborious. Recently, it has been accepted that plants and their products demonstrate one of the main sources for compounds with antimutagenic potential and, indeed, several secondary plant metabolites have demonstrated chemo preventive activity against to genotoxic agents [7]. These antimutagens and anticarcinogens may inhibit one or more stages of the carcinogenic process and prevent or delay the formation of cancer. In addition to all these situations it should be noted that infectious diseases and the increasing antibiotic resistance are major global problems which threaten human health. Due to the indiscriminate usage of commercial antimicrobial drugs, multi-drug resistance in both human and plant pathogenic microorganisms has developed. Therefore scientists have tried to notice new antimicrobial and antimutagenic substances from various sources including plants [8]. It is known that, now natural products and their derivatives hold more than 50% of all the drugs in clinical usage with one quarter originating from higher plants.

The genus *Cotoneaster* (Rosaceae) comprises 85 species, which are predominately distributed around the Europe and Asia [9]. The genus is represented in Turkey by 8 species [10]. Several species of *Cotoneaster* are used to medicinal purposes such as cardiotonic, diuretic, expectorant and antiviral in different countries [11], [12]. *Cotoneaster nummularia*, called “Dağ muşmulası or Tavşan elması”, is perennial herbs and grows in Anatolia. The medicinal uses of the species range from cures for diabetes mellitus and hemorrhoids, to being used as an expectorant in Anatolia folk medicine [13]–[15]. Although this plant has numerous applications on herbal remedy, thus far there are no scientific evidences behind the uses. In view of the above, the goal of this study was to thoroughly examine the composition (especially phenolic components) and biological activities (anti-oxidant, anti-bacterial, mutagenic/anti-mutagenic, anti-cholinesterase, anti-tyrosinase and anti-diabetic (α-amylase and α-glucosidase) of different solvent extracts from *C. nummularia* which is a wild plant.

## Materials and Methods

### Plant Material

*Cotoneaster nummularia* was collected from Konya-Turkey (Yukselen village, dry slopes) Taxonomic identification of the plant material was confirmed by the senior taxonomist Dr. Murad Aydin Sanda, from the Department of Biology, Selcuk University. The voucher specimen has been deposited at the Herbarium of the Department of Biology, Selcuk University, Konya-Turkey.

### Ethics Statement

For the collection of plants, no specific permits were required for the described field studies. For any locations/activities, no specific permissions were required. All locations where the plants were collected were not privately-owned or protected in any way and the field studies did not involve endangered or protected species. This study was approved by the University of Selcuk institutional review board.

### Extraction

To produce solvent extracts, the air-dried samples (10 g) of the twigs of *Cotoneaster nummularia* were extracted with 250 mL of solvents (ethyl acetate or methanol) in a Soxhlet apparatus for 6–8 h. The extracts concentrated under vacuum at 40°C by using a rotary evaporator. For water extract, the air-dried samples (5 g) were boiled with 250 mL of distilled water for 30 min. The water extract were filtered and lyophilized (−80°C, 48 h). Extracts were stored at +4°C in dark until use. Extracts obtained using organic solvents were dissolved in methanol and then filtered. Water extract was dissolved in water at different concentrations.

### Quantification of Phenolic Compounds by RP-HPLC

Phenolic compounds were evaluated by reversed-phase high-performance liquid chromatography (RP-HPLC). Detection and quantification were carried out with a LC-10ADvp pump, a Diode Array Detector, a CTO-10Avp column heater, SCL-10Avp system controller, DGU-14A degasser and SIL-10ADvp auto sampler. Separations were conducted at 30°C on C-18 reversed-phase column (250 mm ×4.6 mm length, 5 µm particle size). The eluates were detected at 278 nm. The mobile phases were A: 3.0% acetic acid in distilled water and B: methanol. For analysis, the samples were dissolved in methanol, and 20 µl of this solution was injected into the column. The elution gradient applied at a flow rate of 0.8 ml/min was: 93% A/7% B for 0.1 min, 72%A/28%B in 20 min, 75%A/25%B in 8 min, 70%A/30%B in 7 min and same gradient for 15 min, 67%A/33%B in 10 min, 58%A/42%B in 2 min, 50%A/50%B in 8 min, 30%A/70%B in 3 min, 20%A/80%B in 2 min 100%B in 5 min until the end of the run. Phenolic compositions of the extracts were determined by a modified method of Caponio *et al*. [16]. Gallic acid, protocatechuic acid, (+)-catechin, p-hydroxybenzoic acid, chlorogenic acid, caffeic acid, (-)-epicatechin, syringic acid, vanilin, p-coumaric acid, ferulic acid, sinapinic acid, benzoic acid, o-coumaric acid, rutin, naringin, hesperidin, rosmarinic acid, eriodictyol, trans-cinnamic acid, quercetin, naringenin, luteolin, kaempferol and apigenin were used as standard. Identification and quantitative analysis were done by comparison with standards. The amount of each phenolic compound was expressed as mg per gram of the extract.

### Determination of Total Bioactive Components

#### Total phenolic content

The total phenolic content was determined by employing the methods given in the literature [17] with slight modification. Sample solution (0.25 mL) was mixed with diluted Folin-Ciocalteu reagent (1 mL, 1∶9) and shaken vigorously. After 3 min, Na_2_CO_3_ solution (0.75 mL, 1%) was added and the sample absorbance was read at 760 nm after 2 h incubation at room temperature. The total phenolic content was expressed as equivalents of gallic acid (mgGAEs/g extract) according to the equation obtained from the standard gallic acid graph.

#### Total flavonoid content

The total flavonoid content was determined using the Dowd method as adapted by Berk *et al*. [18]. Briefly, sample solution (1 mL) was mixed with the same volume of aluminium trichloride (2%) in methanol. Similarly, a blank was prepared by adding sample solution (1 mL) to methanol (1 mL) without AlCl_3_. The sample and blank absorbances were read at 415 nm after 10 min incubation at room temperature. The absorbance of the blank was subtracted from that of the sample. The total flavonoid content was expressed as equivalents of rutin (mgREs/g extract) according to the equation obtained from the standard rutin graph.

### Total Antioxidant Activity

#### Phosphomolybdenum method

The total antioxidant activities of the samples were evaluated by phosphomolybdenum method according to Berk *et al*. [18] with slight modification. Sample solution (0.3 mL) was combined with 3 mL of reagent solution (0.6 M sulfuric acid, 28 mM sodium phosphate and 4 mM ammonium molybdate). The sample absorbance was read at 695 nm after a 90 min incubation at 95°C. The total antioxidant capacity was expressed as equivalents of ascorbic acid (mgAEs/g extract) as determined by the equation obtained from the standard ascorbic acid graph.

#### β-carotene–linoleic acid method

In this assay antioxidant activity is determined by measuring the inhibition of the volatile organic compounds and the conjugated diene hydroperoxides arising from linoleic acid oxidation [19] with slight modification. A stock solution of β-carotene–linoleic acid mixture was prepared as following: 0.5 mg β-carotene was dissolved in chloroform (1 mL, HPLC grade). 25 µL linoleic acid and 200 mg Tween 40 was added. Chloroform was completely evaporated using a vacuum evaporator. Then 100 mL of oxygenated distilled water was added with vigorous shaking; 1.5 mL of this reaction mixture was dispersed to test tubes and sample solution (0.50 mL, 2 mg/mL) were added and the emulsion system was incubated for up to 2 h at 50°C. The same procedure was repeated with the standard (BHA) and a blank. After this incubation period, the sample absorbance was read at 490 nm. Measurement of absorbance was continued until the color of *β*-carotene disappeared. The bleaching rate (R) of *β*-carotene was calculated according to Eq. (1).
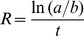
(1)


Where, ln =  natural log, *a* =  absorbance at time 0, *b* =  absorbance at time *t* (30, 60, 90, 120 min). The antioxidant activity (AA) was calculated in terms of percent inhibition relative to the control using Eq. (2).
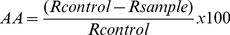
(2)


### Radical Scavenging Activity

#### Free radical scavenging activity (DPPH)

The effects of the samples on 1,1-diphenyl-2-picrylhydrazyl (DPPH) radical were estimated according to Sarikurkcu [20]. Sample solution (1 mL) was added to a 4 ml of a 0.004% methanol solution of DPPH. The sample absorbance was read at 517 nm after a 30 min incubation at room temperature in dark. Inhibition of free radical DPPH in percent (I%) was calculated in following way: (Eq. (3))
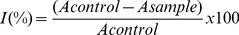
(3)where A_Control_ is the absorbance of the control reaction (containing all reagents except the test compound) and A_Sample_ is the absorbance of the test compound. BHA was used as a control. %50 of free radical inhibition (IC_50_) of samples was calculated. The lower the IC_50_ value indicates high antioxidant capacity.

#### ABTS (2,2Azino-bis (3-ethylbenzothiazloine-6-sulfonic acid)) radical cation scavenging activity

The scavenging activity against ABTS cation radical was measured according to the method of Re *et al*. [21] with slight modification. Briefly, ABTS^.+^ radical cation was produced directly by reacting 7 mM ABTS solution with 2.45 mM potassium persulfate and allowing the mixture to stand for 12–16 in dark at the room temperature. Prior to beginning the assay, ABTS solution was diluted with methanol to an absorbance of 0.700±0.02 at 734 nm. Sample solution (1 mL) was added to ABTS solution (2 mL) and mixed. The sample absorbance was read at 734 nm after 30 min incubation at room temperature. The results were reported as IC_50_.

#### Superoxide anion (O_2_^.−^) radical scavenging activity

The superoxide anion radical scavenging activity was followed in the riboflavin-light-nitroblue tetra zolium (NBT) system [22] with slight modification. Sample solution (0.25 mL) was added to reaction mixture containing riboflavin (0.1 mL, 0.1 mg/mL), EDTA (0.1 mL, 12 mM) and NBT (0.05 mL, 1 mg/mL), phosphate buffer (1 mL, 50 mM, pH 7.8) and 1-butanol (0.5 mL). The reaction mixture was illuminated for 10 min at room temperature and the sample absorbance was read at 560 nm. The unilluminated reaction mixture was used as a blank. The absorbance of the blank was subtracted from that of the sample and the results were reported as IC_50_.

### Reducing Power

#### Cupric ion reducing method (CUPRAC assay)

The cupric ion reducing activity (CUPRAC) was determined according to the method of Apak *et al*. [23]. Sample solution (0.5 mL) was added to premixed reaction mixture containing CuCl_2_ (1 mL, 10 mM), neocuproine (1 mL, 7.5 mM) and NH_4_Ac buffer (1 mL, 1 M, pH 7.0). Similarly, a blank was prepared by adding sample solution (0.5 mL) to premixed reaction mixture (3 mL) without CuCl_2_. Then, the sample and blank absorbances were read at 450 nm after a 30 min incubation at room temperature. The absorbance of the blank was subtracted from that of the sample. The EC_50_ value (the effective concentration at which the absorbance was 0.5) was calculated for extracts and BHA.

#### Reducing power activity (Iron (III) to iron (II) reduction)

The ferric reducing power method applied with slight modifications of the method of Oyaizu [24]. Various concentrations of the extracts (2.5 mL) were mixed with 2.5 mL of 0.2 M phosphate buffer (pH 6.6) and 2.5 mL of 1% potassium ferricyanide. The mixture was incubated at 50°C for 20 min. After 2.5 mL of 10% trichloroacetic acid was added. 2.5 mL of the reaction mixture was mixed with 2.5 mL distilled water and 0.5 mL of 0.1% ferric chloride. The solution absorbance was measured at 700 nm. The results were evaluated by EC_50_ values.

### Metal Chelating Activity on Ferrous Ions

The metal chelating activity on ferrous ions was determined by the method described by Aktumsek *et al*. [25]. Briefly, sample solution (2 mL) was added to FeCl_2_ solution (0.05 mL, 2 mM). The reaction was initiated by the addition of 5 mM ferrozine (0.2 mL). Similarly, a blank was prepared by adding sample solution (2 mL) to FeCl_2_ solution (0.05 mL, 2 mM) and water (0.2 mL) without ferrozine. Then, the sample and blank absorbances were read at 562 nm after 10 min incubation at room temperature. The absorbance of the blank was subtracted from that of the sample. The metal chelating activity was expressed as equivalents of EDTA (mgEDTAEs/g extract) according to the equation obtained from the standard EDTA graph.

### Enzyme Inhibitory Activity

#### Cholinesterase inhibition

Cholinesterase (ChE) inhibitory activity was measured using Ellman's method, as previously reported [25] with slight modification. Sample solution (50 µL) was mixed with DTNB ((5,5-dithio-bis(2-nitrobenzoic) acid) (125 µL) and AChE (acetylcholinesterase) or BChE (butyrylcholinesterase) solution (25 µL) in Tris-HCl buffer (pH 8.0) in a 96-well microplate and incubated for 15 min at 25°C. The reaction was then initiated with the addition of acetyl thiocholine iodide (ATCI) or butyryl thiocholine chloride (BTCl) (25 µL). Similarly, a blank was prepared by adding sample solution to all reaction reagents without enzyme (AChE or BChE) solution. The sample and blank absorbances were read at 405 nm after 10 min incubation at 25°C. The absorbance of the blank was subtracted from that of the sample and the cholinesterase inhibitory activity was expressed as equivalents of galanthamine (mgGALAEs/g extract).

#### Tyrosinase inhibition

Tyrosinase inhibitory activity was measured using the modified dopachrome method with L-DOPA as substrate, as previously reported [26] with slight modification. Sample solution (25 µL) was mixed with tyrosinase solution (40 µL) and phosphate buffer (100 µL, pH 6.8) in a 96-well microplate and incubated for 15 min at 25°C. The reaction was then initiated with the addition of L-DOPA (40 µL). Similarly, a blank was prepared by adding sample solution to all reaction reagents without enzyme (tyrosinase) solution. The sample and blank absorbances were read at 492 nm after a 10 min incubation at 25°C. The absorbance of the blank was subtracted from that of the sample and the tyrosinase inhibitory activity was expressed as equivalents of kojic acid (mgKAEs/g extract).

#### α-amylase inhibition

α-amylase inhibitory activity was performed using Caraway-Somogyi iodine/potassium iodide (IKI) method [27] with some modifications. Sample solution (25 µL) was mixed with α-amylase solution (50 µL) in phosphate buffer (pH 6.9 with 6 mM sodium chloride) in a 96-well microplate and incubated for 10 min at 37°C. After pre-incubation, the reaction was initiated with the addition of starch solution (50 µL, 0.05%). Similarly, a blank was prepared by adding sample solution to all reaction reagents without enzyme (α-amylase) solution. The reaction mixture was incubated 10 min at 37°C. The reaction was then stopped with the addition of HCl (25 µL, 1 M). This was followed by addition of the iodine-potassium iodide solution (100 µL). The sample and blank absorbances were read at 630 nm. The absorbance of the blank was subtracted from that of the sample and the α-amylase inhibitory activity was expressed as equivalents of acarbose (mgACAEs/g extract).

#### α-glucosidase inhibition

α-glucosidase inhibitory activity was performed by the previous method [27] with some modifications. Sample solution (50 µL) was mixed with glutathione (50 µL), α-glucosidase solution (50 µL) in phosphate buffer (pH 6.8) and PNPG (4-N-trophenyl-α-D-glucopyranoside) (50 µL) in a 96-well microplate and incubated for 15 min at 37°C. Similarly, a blank was prepared by adding sample solution to all reaction reagents without enzyme (α-glucosidase) solution. The reaction was then stopped with the addition of sodium carbonate (50 µL, 0.2 M). The sample and blank absorbances were read at 400 nm. The absorbance of the blank was subtracted from that of the sample and the α-glucosidase inhibitory activity was expressed as equivalents of acarbose (mgACAEs/g extract).

### Mutagenicity/Antimutagenicity Evaluation

#### Strains

The *Salmonella typhimurium* test strains TA98 and TA 100 were obtained from Microbiology Research Laboratory, Science Faculty, Selcuk University. While TA98 were used for determining the frame shift, TA100 was used to determine the base pair exchange type of mutations.

#### Determination of toxic dose levels

Cytotoxicity of the *Cotoneaster* extract was stated by the method of Dean *et al* [28]. Non cytotoxic doses (10000, 5000, 1000, 100, 10 µg/plate) of the extract was used in the experiments.

#### Mutagenicity assay

The Ames test was performed as a standard plate incorporation assay with *S. typhimurium* strains TA98 and TA100 in the presence or absence of S9 mix [29]. Selection of the strains was based on the testing and strain selection strategies of Mortelmans and Zeiger [30]. These strains were tested on the basis of associated genetic markers. For each tester strain, a specific positive control was always used to test the experimental flaws, if any. While 4-nitro-*O*-phenylenediamine (4-NPDA, 20 µg/plate) for TA 98 and sodium azide (SA, 5 µg/plate) for TA100 were used as positive controls without S9 mix (metabolic activation enzymes), 2-aminofluorene (2-AF,) and 2-aminoanthracene (2-AA, 20 µg/plate) were used as positive controls with S9, respectively.

S9 mix (500 µL) (or 500 µL phosphate buffer), the test solution (100 µL) for each concentration and a cell suspension (100 µL) from an overnight culture (1−2×10^9^ cells/mL) were added to 2.5 mL top agar (kept at 45°C) and vortexed for eight seconds. The entire mixture was overlaid on the minimal agar plate. The plates were incubated at 37°C for 72 h and then the revertant bacterial colonies on each plate were counted. Both the positive and negative controls (distilled water) were maintained concurrently. Samples were tested on triplicate plates in two independent parallel experiments.

#### Antimutagenicity assay

A 100 µL aliquot of bacterial suspension including 1−2×10^9^ bacteria, 100 µL of different concentrations of extract, 100 µL of positive mutagen solution, 500 µL of S9 mixture or phosphate buffer (0.1 M) were added to 2.5 mL of top agar containing 10 % of histidine/biotin (0.5 mM) for both *Salmonella* strains, mixed by vortex for eight seconds and poured onto the surface of minimal glucose agar plaque. In the antimutagenicity test, the inhibitions of mutagenic activities of 4-NPDA without S9 metabolic activation and 2-AF with S9 for *S. typhimurium* TA 98, SA in the absence of S9 mix and 2-AA in the presence of S9 mix for *S. typhimurium* TA 100 by the plant extract samples were determined. His^+^ revertants were counted after incubation of the plates at 37°C for 48–72 h. Each sample was assayed using triplicate plates and the data presented as mean ± SD of two independent assays. The number of revertant colonies grown on plates containing the mutagen without plant extract was defined as 100% with 0% inhibition. The percentage of inhibition was calculated according to the formula: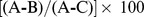, where A  =  No. of his. revertants in the absence of sample, B  =  No. of his. revertants in the presence of sample, C  =  spontaneous revertants [31]. Inhibition rate of 40% or more was defined as strong antimutagenicity, 25–40% inhibition as moderate antimutagenicity. Inhibitory effects of less than 25% were considered as weak and were not recognized as a positive result [32].

### Antibacterial Assay

Strains of *Escherichia coli* ATCC 25922, *Bacillus cereus* ATCC 11778, *Pseudomonas aeruginosa* ATCC 27853, methicillin sensitive *Staphylococcus aureus* ATCC 25923 (MSSA), *Klebsiellapneumoniae* ATCC 70603, *Salmonella enteritidis* ATCC 13076, *Streptococcus pneumoniae* ATCC 10015, *Sarcinalutea* ATCC 9341, *Enterococcus faecalis* ATCC 29212, methicillin resistant *Staphylococcus aureus* ATCC 43300 (MRSA) for the determination of antibacterial activities, and strains of methicillin resistant *Staphylococcus aureus* isolated from the clinical samples, for the detection of anti-MRSA activities of *Cotoneaster* extracts were used. The methicillin resistances of strains were determined by agar screening, oxacillin disc diffusion and broth microdilution methods. The standard microorganisms were got access to from Microbiology Research Laboratory, Department of Biology, Selcuk University, and Konya, Turkey. Standard and isolated strains of bacteria were grown to exponential phase in Brain-Heart Infusion Broth at 37°C overnight with aeration. The cultures were then plated on Mueller-Hinton Agar, overnight at 37°C with aeration. Then, approximately five colonies of cultures suspended in sterile physiologic water. The bacterial suspensions were adjusted to 0.5 McFarland standard turbidity (10^8^ CFU/ml). Finally, these suspensions used as inoculums were prepared at 10^5^ CFU/ml by diluting fresh cultures at McFarland 0.5 density. The broth microdilution method was employed for antibacterial and anti-MRSA activity tests. Mueller-Hinton Broth (100 µl) was placed into each 96 wells of microplates. Extract solutions initially prepared at a concentration of 10 mg/ml were added into first wells of microplates and two fold dilutions of the extracts (2.5–0.00122 mg/ml) were made by dispensing the solutions to the remaining wells. Then, 100 µl of culture suspensions were inoculated to each well. Gentamicin and Oxacilin were used as positive control. The sealed microplates were incubated at 35°C for 18 h. Microbial growth was determined by adding 20 µL of 2,3,5-Triphenyl-tetrazolium chloride (0.5%) after incubation to each well and incubating for 30 minute at 37oC [33]. The lowest concentration of the extracts that completely inhibit macroscopic growth was determined as minimum inhibitory concentrations (MICs).

### Statistical Analysis

For all the experiments all the assays were carried out in triplicate. The results are expressed as mean values and standard deviation (SD). The differences between the different extracts were analyzed using one-way analysis of variance (ANOVA) followed by Tukey's honestly significant difference post hoc test with α = 0.05. This treatment was carried out using SPSS v. 14.0 program.

## Results and Discussion

### Antioxidant Potentials and Phytochemical Composition

Antioxidant capacities of ethyl acetate, methanol and water extracts from *C. nummularia* were evaluated by free radical scavenging assay (DPPH, ABTS and O_2_), reducing power, phosphomolybdenum, β-carotene/linoleic acid bleaching and metal chelating assays. Free radical scavenging activities expressed as IC_50_ values and the results are summarized in Table 1. BHA was the reagent used as standard in these assays. Generally, methanol and water extracts showed the highest activity with lower values of IC_50_. Occurrence of the highest DPPH and O_2_ scavenging activities were observed in the water extract of the plant, followed by the methanol extract. However, the greatest ABTS inhibition was caused by the methanol extract (IC_50_:0.020 mg/ml) closely followed by the water extract (IC_50_:0.023 mg/ml). There were no significant differences in free radical scavenging activities between methanol and water extracts (*p*>0.05). As evidences by this table, ethyl acetate extract showed lowest activity with IC_50_ values two times higher than methanol and water extracts. However, BHA has a stronger effect of scavenging free radicals than tested extracts in the test systems.

**Table 1:** Antioxidant activities of three extracts from *Cotoneaster nummularia* (mean±S.D).

	Free Radical Scavenging (IC_50_ (mg/ml)			Reducing Power (EC_50_ (mg/ml)		Total Antioxidant Capacity		Metal Chelating Activity
Solvent	DPPH	ABTS	O_2_ -	Ferric Reducing Power	CUPRAC	Phosphomolybdenum (mgAEs/g)*	β-carotene/linoleic acid assay (%)	Ferrous ion chelating (mgEDTAEs/g)**
Ethyl acetate	0.252±0.004a^b^	0.043±0.001a	1.603±0.121a	0.654±0.006a	0.263±0.001a	56.06±2.76c	85.46±0.08d	0.25±0.01c
Methanol	0.104±0.005bc	0.020±0.001cd	1.094±0.061bc	0.149±0.039bc	0.022±0.007d	161.26±4.83b	89.63±1.70c	4.45±0.01b
Water	0.097±0.001c	0.023±0.001bc	1.066±0.021c	0.143±0.03c	0.044±0.005bc	177.29±1.75ab	93.04±0.55bc	18.66±0.43a
BHA	0.016±0.001d	0.015±0.001d	0.226±0.016d	0.055±0.001d	0.028±0.001cd	nt	95.07±0.75ab	nt

b In same column marked with different letters indicate significant difference (p<0.05).

*AEs: ascorbic acid equivalents.

**EDTAEs: EDTA equivalents.

nt: no tested.

Reducing power activities were evaluated by using EC_50_ (the effective concentration at which the absorbance was 0.5). Reducing powers of tested extracts appeared to be in the same manner with free radical scavenging activity (Table 1). In the ferric and cupric reducing power assays, the methanol and water extracts were the most effective even the methanol extract a lower EC_50_ value in the CUPRAC assay than that of the standard antioxidant (BHA). Here, ferric reducing activities of methanol and water extracts were not significantly different (*p*>0.05), but these extracts showed significant difference in cupric reducing activity (*p*<0.05). These results are in accord with those reported by several investigators who indicated that methanol and water extracts the most active in free radical and reducing power assays [34], [35]. Ethyl acetate extract has the lowest activity in both ferric and cupric reducing power assays (*p*<0.05).

Total antioxidant activities of tested extracts were investigated by phosphomolybdenum and β-carotene/linoleic acid assays. Phosphomolybdenum method is based on the reduction of Mo (IV) to Mo (V) by the antioxidants and the subsequent formation of green phosphate/Mo (V) compounds with a maximum absorption at 695 nm. In this assay, the highest activity was demonstrated by water extract (177.2 mgAEs/g) followed by methanol (161.26 mgAEs/g) and ethyl acetate extract (56.06 mgAEs/g). Apparently, the total antioxidant activity of water extract was 3.16 fold higher than that of ethyl acetate (Table 1). However, differences in the ascorbic acid equivalent values of methanol and water extracts were not significant (*p>*0.05) Our results are concomitant with previous findings where superior total antioxidant activities in water extracts of several plants compared to other extracts was reported [36], [37]. The potential of the extracts to inhibit lipid peroxidation was evaluated using the β-carotene/linoleic acid bleaching assay. In terms of inhibition ability of linoleic acid oxidation, those samples can be ranked from high to low in the following order: BHA (95.07%)> Water (93.04%)> Methanol (89.63%)> Ethyl acetate (85.46%). Nonetheless, inhibition values of methanol and water extracts were not statistically different (*p*>0.05). Water extract exhibited marked antioxidant ability close to of BHA. Hence, the water extract could be considered as a natural inhibitor for prevention of lipid oxidation in food industry. It is probable that the antioxidant components such as phenolics in the water extract can reduce the extent of β-carotene destruction by inhibiting the linoleic acid oxidation in the test system.

Metal ion chelating activity of each extract was tested against ferrous ion. The chelating activities of the extracts were evaluated using EDTA as a standard (mg EDTAs/g extract). In accordance with results of other antioxidant assays, potent chelation abilities were again detected in the water extracts with 18.66 mg EDTA s/g (Table 1) (*p*<0.05). Since ferrous ions were the most effective pro-oxidant for lipid peroxidation, the water extract from *C. nummularia* would be beneficial. In fact, numerous studies indicated that some plant extracts are capable of with and stabilizing ferrous ion and rendering it's unable to participate in metal-catalyzed initiation and hydroperoxide decomposition reactions [27], [38], [39].

**Figure 1:**
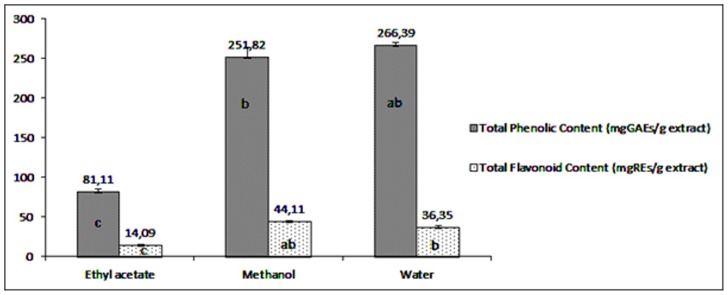
Total phenolic and flavonoid content in three extracts from *Cotoneaster nummularia* ((mean±SD). In each bar different letters indicate significant difference (p<0.05). (GAEs: Gallic acid equivalents: REs: Rutin equivalents).

Total phenolic contents of the *Cotoneaster* extracts ranged from 81.11 to 266.39 mgGAEs/g extracts. Water extract had the highest total phenolics, followed by methanol and ethyl acetate. Similarly, total flavonoid contents were found to be significantly high in water and methanol extracts. Differences in the total phenolic and flavonoid content of methanol and water extracts were not significant (*p*>0.05). Based on these results, the flavonoid content of methanol extract was three times higher than that on the ethyl acetate extract (Figure 1) (*p*<0.05). The results of total phenolic content showed a similar tendency to those of the antioxidant abilities. In this direction, the high content of total phenolics in methanol and water extracts might be explain the strong antioxidant abilities of the extracts. These results are in accordance with other reports in the literature, which showed strong relationship between antioxidant activities and total phenolic content [40]–[42].

**Figure 2:**
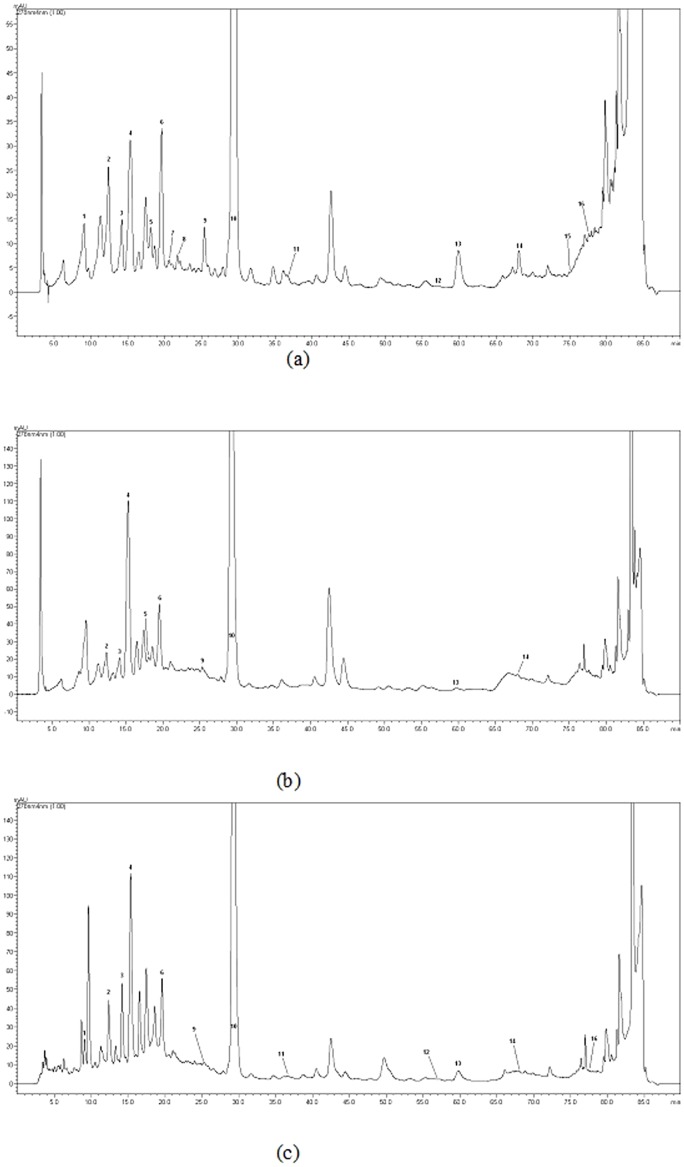
HPLC chromatograms of the extracts from *Cotoneaster nummularia* (a: ethyl acetate, b: methanol and c: water). [1. Protocatechuic acid, 2. (+)-Catechin, 3. *p*-Hydroxybenzoic acid, 4. Chlorogenic acid, 5. Caffeic acid, 6. (-)-Epicatechin, 7. Syringic acid, 8. Vanilin, 9. *p*-Coumaric acid, 10. Ferulic acid, 11. Benzoic acid, 12. Rosmarinic acid, 13. Eriodictyol, 14. *trans*-Cinnamic acid, 15. Luteolin, 16. Apigenin]

Although a number of analytical methods have been proposed for the separation and determination of phenolic compounds, high performance liquid chromatography (HPLC) technique with diode array detector has become a dominant procedure since it has some advantages such as simple sample treatment, short analysis time and high reproducibility [43]. Using HPLC analysis, the content of the principal phenolic compounds of plant extracts are listed in Table 2. 25 standard phenolics were analyzed and 16 of them were identified in the extracts (Figure 2). Gallic acid, quercetin, naringenin, hesperidin, sinapic acid, *o*-coumaric acid, rutin, naringin and kaempferol were not detected in these extracts (data is not shown in Table 2). Major phenolic compounds were determined as ferulic acid (22.60–28.36 mg/g extract), chlorogenic acid (5.70–16.66 mg/g extract), -(-) epicatechin (5.24–6.92 mg/g extract) and catechin (3.26–6.14 mg/g extract). These data established that the biological activities of *C. nummularia* could be attributed to their polyphenol compounds. Recent studies showed that ferulic acid acts as a potent antioxidant by scavenging free radicals. In addition, ferulic acid proposed as a potential treatment for many disorders including Alzhemier's diseases, diabetes mellitus and skin diseases [44]–[46]. Again, it possesses a wide spectrum of antimicrobial (Gram-positive bacteria, Gram-negative bacteria and yeasts) and antimutagenic activity [47], [48]. Thus, high concentration of ferulic acid was thought to be responsible for the biological activities exerted. Actually, the higher ferulic acid content in methanol extract conferred to its strong biological activities. Moreover, chlorogenic acids, (+)-catechin and (-)-epicatechin content were closely correlated to biological activities such as metal chelating and inhibition of lipid peroxidation [49]. In this way, the superiority of methanol and water extracts biological activities may be explained by the amount and nature of these major phenolic compounds.

**Table 2:** Phenolic components in the solvent extracts from *Cotoneaster nummularia* (mg/g extract) (mean±SD).

No	Phenolic Components	Ethyl Acetate	Methanol	Water
**1**	Protocatechuic acid	1.64±0.04a^a^	nd	1.16±0.04b
**2**	(+)- Catechin	6.14±0.16a	4.58±0.14b	3.26±0.12c
**3**	*p-*Hydroxybenzoic acid	1.08±0.04b	0.94±0.04b	3.16±0.04a
**4**	Chlorogenic acid	5.70±0.12c	16.66±0.26a	13.92±0.24b
**5**	Caffeic acid	0.54±0.01a	0.56±0.01a	nd
**6**	(-)- Epicatechin	5.24±0.32a	6.50±0.32a	6.92±0.32a
**7**	Syringic acid	0.12±0.01	nd	nd
**8**	Vanilin	0.08±0.01	nd	Nd
**9**	*p-* Coumaric acid	0.26±0.02b	0.48±0.01a	0.18±0.01c
**10**	Ferulic acid	25.54±0.62ab	28.36±0.62a	22.60±0.62b
**11**	Benzoic acid	1.32±0.04a	nd	1.28±0.04a
**12**	Rosmarinic acid	0.14±0.01b	nd	0.28±0.01a
**13**	Eriodictyol	1.16±0.01a	0.28±0.01c	1.00±0.01b
**14**	*trans*-Cinnamic acid	0.16±0.02b	0.24±0.02a	0.02±0.01c
**15**	Luteolin	0.08±0.01	nd	nd
**16**	Apigenin	0.22±0.01a	nd	0.04±0.01b

aIn same row marked different letters indicate significant difference (p<0.05); nd, not determined.

### Enzyme Inhibitory Activities

The ethyl acetate, methanol and water extracts were tested for AChE, BChE, tyrosinase, α-amylase and α-glucosidase inhibitory activities using colorimetric methods in a 96-well microplate. These activities were expressed as equivalents of standard inhibitors (galatamine for AChE and BChE, kojic acid for tyrosinae and acarbose for α-amylase and α-glucosidase). The results are shown in Table 3. The methanol and water extracts exhibited remarkable AChE inhibitory activity with 4.30 and 4.77 mgGALAEs/g extract, respectively. However; water extract had the lowest inhibitory activity on BChE. With regard to tyrosinase inhibitory activity, water extract showed a good inhibitory activity with 32.31 mg KAEs/g. The tyrosinase inhibitory activity of ethyl acetate (24.22 mg KAE/g) is slightly different from that of the methanol extract (24.01 mg KAEs/g). Methanol extract possessed very good inhibitory activity against α-glucosidase and α-amylase. In α- glucosidase inhibition assay the activity was methanol > water > ethyl acetate. In contrast to α- glucosidase assay, the lowest α-amylase inhibition activity was revealed by water extract. To summarize, methanol and water extracts had the higher inhibitory activity on tested enzymes compared to ethyl acetate extract. The effective inhibitory activities of these samples were most likely due to their high phenolic content, especially ferulic acid. Similar results were reported for *Impatiens bicolor* and selected Lamiacae species by Shahwar *et al*. [50] and Vladimir-Knezevic *et al*. [51], respectively. Many phenolic compounds (ferulic acid, catechin eg.) were reported to protect against the above mentioned public health problems [52], [53]. On the other hand, a number of studies were figured that no correlation was between phenolic content/phenolic components and enzyme inhibitory activities [54], [55]. This situation can be explained the complex nature of phytochemicals and their synergistic or antagonistic effects. The present study reports, for the first time, on the *in vitro* enzyme inhibitory activities of the different extracts of *Cotoneaster nummularia*.

**Table 3:** Enzyme inhibitory activities of three extracts from *Cotoneaster nummularia* (mean±SD).

Solvents	AChE (mgGALAEs/g)*	BChE (mgGALAEs/g)*	α-amylase (mgACAEs/g)**	α- Glucosidase (mgACAEs/g)**	Tyrosinase (mgKAEs/g)***
**Ethyl Acetate**	4.07±0.40a^b^	5.46±0.32a	7.91±0.90b	51.02±4.09b	24.22±1.45a
**Methanol**	4.30±0.45a	6.03±0.11a	13.62±1.90a	82.34±0.90a	24.01±1.53a
**Water**	4.77±0.26a	0.65±0.01b	1.90±0.88c	69.54±4.21c	32.31±0.26b

b In same column marked with different letters indicate significant difference (p <0.05).

*GALAEs: galanthamine equivalents.

**ACAEs: acarbose equivalents.

***KAEs: kojic acid equivalents.

### Anti-bacterial Activity

In this study antibacterial and anti-MRSA activities of water, methanol and ethyl acetate extracts of *Cotoneaster* were investigated by broth microdilution method according to Koç *et al*. [33]. The obtained results are presented in Table 4.

**Table 4:** MIC values of *Cotoneaster* extracts against standard bacteria and MRSA strains isolated from clinical samples.

Tested Microorganisms	MIC values of *Cotoneaster* Extracts (µg/ml)			MIC value of Gentamicin (µg/ml)	MIC value of Oxacillin (µg/ml)
	Ethyl acetate	Methanol	Water		
***Escherichia coli*** ** ATCC 25922**	2500	2500	625	2.44	
***Bacillus cereus*** ** ATCC 11778**	2500	-	-	9.76	
***Pseudomonas aeruginosa*** ** ATCC 27853**	2500	2500	-	9.76	
***Staphylococcus aureus*** ** ATCC 25923 (MSSA)**	2500	2500	625	2.44	0.25
***Klebsiella pneumoniae*** ** ATCC 70603**	2500	-	-	2.44	
***Staphylococcus aureus*** ** ATCC 43300 (MRSA)**	2500	2500	625	78.12	64
***Salmonella enteritidis*** ** ATCC 13076**	2500	-	-	4.88	
***Streptococcus pneumoniae*** ** ATCC 10015**	2500	625	-	2.44	
***Sarcina lutea*** ** ATCC 9341**	2500	-	625	4.88	
***Enterococcus faecalis*** ** ATCC 29212**	2500	312.5	39	2.44	
**MRSA strain 1 (ES 67)**	2500	-	625	156.25	32
**MRSA strain 2 (ES 16)**	2500	-	625	156.25	16
**MRSA strain 3 (ES 107)**	2500	2500	625	156.25	8
**MRSA strain 4 (ES 128)**	2500	2500	625	78.12	≥128
**MRSA strain 5 (ES 123)**	2500	2500	625	78.12	16
**MRSA strain 6 (ES 68)**	2500	2500	625	156.25	≥128
**MRSA strain 7 (ES 124)**	2500	1250	625	78.12	≥128
**MRSA strain 8 (ES 110)**	1250	625	625	156.25	≥128
**MRSA strain 9 (ES 100)**	2500	2500	625	78.12	≥128
**MRSA strain 10 (ES 75)**	2500	-	1250	156.25	≥128
**MRSA strain 11 (ES 29)**	2500	2500	2500	312.5	32
**MRSA strain 12 (ES 69)**	2500	-	2500	312.5	≥128
**MRSA strain 13 (ES 25)**	2500	2500	2500	312.5	≥128
**MRSA strain 14 (ES 93)**	2500	312.5	625	78.12	≥128

Water extract was found to be remarkable antibacterial against gram positive microorganisms. The MIC values were determined as 0.625 mg/ml for *S. aureus* (MSSA), *S. aureus* (MRSA), and *S. lutea*. MSSA, MRSA and *S. lutea* were affected by control antibiotic at concentrations of 0.00244, 0.07812 and 0.00244 mg/ml, respectively. It has been seen that water extract revealed a significant effect against MRSA. While *E. faecalis* was the most sensitive bacterium, *B. cereus* and *S. pneumonia* were resistant Gram-positive bacteria against water extract. The MIC value of water extract was determined as 0.039 mg/ml against *E. faecalis*. Although *E. coli* was affected by water extract at a 0.625 mg/ml dose, *K. pneumoniae, S. enteritidis*, and *P. aeruginosa* were found to be resistant to this extract. As can be clearly seen from the Table 4 Gram-negative microorganisms were more resistant than Gram-positive bacteria against water extract of *Cotoneaster*.

Methanol extract exhibited significant antibacterial activity against *E. faecalis* at a concentration of 0.312 mg/ml. The MIC values of methanol extracts were determined as 2.5 mg/ml against *E. coli, P. aeruginosa*, MSSA, and MRSA. *B. cereus, K. pneumoniae, S. lutea*, and *S. enteritidis* were not affected by this extract at all test doses. The MIC value was determined as 0.625 mg/ml for *S. pneumoniae*. While *P. aeruginosa* and *S. pneumoniae* were found to be resistant to water extract, they were affected by methanol extract. However, MIC values of the water extract were lower than those of methanol extract. Except for MRSA strain 8, the ethyl acetate extract of *Cotoneaster* exhibited antimicrobial activity at a concentration of 2.5 mg/ml against both standard and isolated bacteria tested in the present study. The MIC value was determined as 1.25 mg/ml for MRSA strain 8. It was concluded that *E. faecalis* was the most sensitive bacteria and *B. cereus, K. pneumoniae*, and *S. enteritidis* were the most resistant bacteria against *Cotoneaster* extracts tested except for ethyl acetate extract.

### Anti-methicillin Resistant *Staphylococcus aureus* (MRSA) Activity

The extracts of *Cotoneaster* were tested against clinical isolates of MRSA. MIC values of the extracts obtained from the study against MRSA strains presented in Table 4. The *Cotoneaster* extracts displayed antimicrobial activity against both *S. aureus* ATCC 43300 and all of the14 tested MRSA strains as shown by the MIC values in the broth microdilution method. Especially, water extract of *Cotoneaster* exhibited significant anti MRSA activity at doses of 0.625 mg/ml against MRSA strains from 1 to 9 and strain 14. Gentamicin revealed antimicrobial activity ranging between 0.156 mg/ml–0.078 mg/ml doses against MRSA isolates. The MIC values of the MRSA strains from 10 to 13 were determined as 2.5 mg/ml. It was determined that clinical isolates of MRSA significantly affected from *Cotoneaster* water extracts. The methanol extracts of *Cotoneaster* showed anti MRSA activity at a dose of 2.5 mg/ml against MRSA isolates 3, 4, 5, 6, 9, 11 and 13. Although MRSA strain 14 was the most sensitive bacterium at a dose of 0.312 mg/ml, strains of 1, 2, 10 and 12 was the most resistant bacteria among the clinical isolates against the methanol extract. Our results revealed that ethyl acetate extract of plant had antibacterial and anti MRSA activities at a dose of 2.5 mg/ml except for MRSA strain 8.

### Mutagenic/Antimutagenic Activity

In this study, the mutagenic and antimutagenic activities of *Cotoneaster* water extract (with doses of 10000 µg/plate and lower) were investigated. The assays were performed using the standard plaque incorporation method defined by Maron and Ames [29]. The revertant colony numbers observed in the mutagenicity assay were determined and are given in Table 5.

**Table 5:** Mutagenicity of *Cotoneaster* extract towards *S. typhimurium* TA98 and TA100 strains with or without S9.

	Concentration (µg/plate)	Number of His^+^ Revertants/plate			
		TA 98		TA 100	
		S9 (−)	S9 (+)	S9 (−)	S9 (+)
* **Negative Control**	**100 µl/plate**	22±1 ^a^	40±8 ^a^	118±6 ^a^	115±6 ^a^
® **Positive Control**		744±98 ^b^	4803±109 ^b^	1855±177 ^b^	3878±159 ^b^
***COTONEASTER*** ** WATER EXTRACT**	**0**	26±4 ^a^	40±5 ^a^	122±9 ^a^	115±6 ^a^
	**10000**	26±3 ^a^	45±7 ^a^	123±8 ^a^	132±9 ^a^
	**5000**	25±1 ^a^	47±2 ^a^	124±5 ^a^	117±18 ^a^
	**1000**	22±2 ^a^	53±7 ^a^	125±7 ^a^	123±5 ^a^
	**100**	25±6 ^a^	55±7 ^a^	120±10 ^a^	119±1 ^a^
	**10**	24±4 ^a^	55±3 ^a^	110±23 ^a^	109±16 ^a^

ab Differences between groups having the same letter in the same column are not statistically significant (ANOVA, Tamhane, *p*>0.05).

*Negative control: Sterile distilled water (100 µl/plate) was used as negative control for *S. typhimurium* TA98 and TA100 both in the presence and absence of S9.

® Positive controls:

2-Aminofluorene (5 µg/plate) was used as positive indirect mutagen in the presence of S9 mix; 4-nitro-*O*-fenilendiamine (20 µg/plate) was used as positive direct mutagen in the absence of S9 mix for *S. typhimurium* TA98 strain.

2-Aminoanthracene (20 µg/plate) was used as positive indirect mutagen in the presence of S9 mix; Sodium azide (5 µg/plate) was used as positive direct mutagen in the absence of S9 mix for *S. typhimurium* TA100.

As seen in Table 5, spontaneous revertants were within normal values in all strains examined. The average revertant colony numbers in negative control were 40±8 for TA98 and 115±6 for TA100 with S9 and 22±1 and 118±6 without S9, respectively (*p*>0.05). While application of S9 in TA98 was increased revertant colony numbers, application of S9 in TA100 was decreased revertant colony numbers (*p*>0.05). On the contrary, the plates with the positive control mutagens (SA, 2-AF, 2-AA and 4-NPDA) showed significant increases relative to the spontaneous mutation rate in the two tested strains. Most of the results, increasing or decreasing relative to negative control group, were not statistically significant at *p*<0.05 (Tamhane) in examined strains. In order to establish a dose-response relationship, 5 different concentrations of *Cotoneaster* extract were tested, and no induced revertants were observed along the dose range tested in either with or without S9 with two strains. The results of the present study showed that all test doses of *Cotoneaster* extract were not found to be mutagenic for *S. typhimurium* TA98 and TA100 in the presence and absence of S9 mix.

The revertant colony numbers observed in the antimutagenicity assay and inhibition (%) rates of the extracts were given in Table 6. *Cotoneaster* extract exhibited moderate antimutagenic activities at doses of 10000, 5000 and 1000 µg (32%, 33%, and 31%, respectively) against 4-NPDA in the absence of S9 mix in *S. typhimurium* TA98 (Table 6). It was determined that there was dose-response relationships between concentrations tested (*p*<0.05). Induced inhibition ratios were observed along the dose range tested in the absence of S9 mix. On the other hand, 100 and 10 µg doses of the extract were found to be weak antimutagenic with a ratio of 19% and 20%, respectively. While *Cotoneaster* extracts showed strong antimutagenicity at doses of 10000 (50%) and 5000 µg (49%) against 2-AF; 1000, 100 and 10 µg doses of the extract exhibited moderate antimutagenic activities in the presence of S9 mix in TA98 strain with a ratio of 40%, 29%, and 25%, respectively (*p*<0.05). It was determined that metabolic activation enzymes (S9 mix) induced the inhibition ratios of the extract compared to those of extracts in the absence of S9 and a dose-response relationship was observed along the dose range tested.

**Table 6:** Antimutagenicity and inhibition rates of *Cotoneaster* extracts towards *S. typhimurium* TA98 and TA100 strains with and without metabolic activation (S9).

	Concentration (µg/plate)	Number of His^+^ Revertants/plate							
		TA 98				TA 100			
		S9 (−)	% inhibition	S9 (+)	% inhibition	S9 (−)	% inhibition	S9 (+)	% inhibition
* **Negative Control**	**100 µl/plate**	48±4 ^a^		40±8 ^a^		146±4 ^a^		115±6 f	
® **Positive Control**		849±41 d	0	4803±109 ^e^	0	2546±142 ^c^	0	3878±159 ^g^	0
***COTONEASTER*** ** WATER EXTRACT**	**0**	48±5 ^a^		40±5 ^a^		140±9 ^a^		114±16 ^f^	
	**10000**	607±38 ^b^	32	2425±256 ^b^	50	1586±159 ^b^	40	1674±224 ^fh^	59
	**5000**	603±25 ^b^	33	2471±91 ^bc^	49	1641±340 ^b^	38	1795±328 fgh	55
	**1000**	618±29 ^b^	31	2906±74 ^c^	40	1685±293 ^b^	36	1670±300 ^fh^	58
	**100**	717±28 ^c^	19	3417±274 ^d^	29	1900±334 ^b^	27	3493±219 ^g^	10
	**10**	710±49 ^c^	20	3633±192 ^d^	25	2060±225 ^bc^	20	3214±95 ^gh^	18

abcde Differences between groups having the same letter in the same column are not statistically significant (ANOVA, Tukey HSD, *p*>0.05).

fgh Differences between groups having the same letter in the same column are not statistically significant (ANOVA, Tamhane, *p*>0.05).

*Negative control: Sterile distilled water (100 µl/plate) was used as negative control for *S. typhimurium* TA98 and TA100 both in the presence and absence of S9.

® Positive controls:

2-Aminofluorene (5 µg/plate) was used as positive indirect mutagen in the presence of S9 mix; 4-nitro-*O*-fenilendiamine (20 µg/plate) was used as positive direct mutagen in the absence of S9 mix for *S. typhimurium* TA98 strain.

2-Aminoanthracene (20 µg/plate) was used as positive indirect mutagen in the presence of S9 mix; Sodium azide (5 µg/plate) was used as positive direct mutagen in the absence of S9 mix for *S. typhimurium* TA100.

It was seen that *Cotoneaster* extract manifested moderate antimutagenicity at concentrations of 10000, 5000, 1000, and 100 µg (40%, 38%, 36%, and 27%, respectively) against SA, while 10 µg dose of extract was found to be weak antimutagenic with a ratio of 20% in the absence of S9 mix in TA100 strain (Table 6). Except for 100 and 10 µg, all doses tested exhibited strong antimutagenic activity against 2-AA in the presence of metabolic activation system (Table 6). The highest inhibition ratio (59%) was observed in 10000 µg/plate dose of the extract, followed by 1000 µg (58%) and 5000 µg (55%). Meanwhile, the extracts at concentrations of 100 and 10 µg were found to be weak antimutagenic capacities with S9 in TA 100 strain.

Based on the results it was determined that *Cotoneaster* water extract had significant antimutagenic capacity in the presence of metabolic activation enzymes (S9) for TA98 at concentrations of 10000, 5000,1000, and 100 µg/plate against 2-AF, for TA100 strain at concentrations of 10000, 5000,1000 µg/plate against 2-AA.

Overall, it can be stated that the S9 enzymes increase the antimutagenic activities of *Cotoneaster* extracts significantly (Table 6). A possible cause of this can be explained by the following way: The antimutagenic response is activated by invoking the competitive inhibition by liver glycosides of P450 isoenzymes [56]. It has been previously determined that some plant metabolites are potent inhibitors of cytochrome c (P450) reductase [57]. Kappus [58] demonstrated that this preventive activity after metabolic activation is related to the function of cytochrome P450 isoforms in detoxification systems with reductase or oxygenase, whose function in the system is antioxidant scavenging, neutralizing compounds that generate oxygen radicals, free radicals and reactive oxygen species [59]. Thus, the greatest antimutagenic activity observed in assays in the presence of a metabolic fraction may be related to the activation of a cytochrome P450 which mediates the oxidation of promutagens [60], indicating that this action could be due to the competitive inhibition by glycosides of cytochrome P450, thus avoiding formation of the promutagen [56]; [61]. This plant may be natural source of antimutagenic agents and may be used in the pharmacology industry.

## Conclusions

To sum up, such detailed studies on chemical composition and biological activity of different extracts from *C. nummularia* have been conducted for the first time. Thus their chemical composition was determined indicating significant amounts of polyphenols, ferulic acid in particular. Based on biological activity assays, anti-oxidant, anti-bacterial, mutagenic/anti-mutagenic, anti-cholinesterase, anti-tyrosinase, anti-amylase and anti-glucosidase of the extracts were revealed. Water and methanol extracts possess notable biological properties. A high positive correlation observed between phytochemical composition and biological activities. Moreover, the water extract showed no mutagenic effect when tested with Ames test. Our data showed that there was no uniform response within or between the bacterial strains in terms of susceptibility to antimicrobial compounds in the water, methanol, and ethyl acetate extracts of *Cotoneaster*. These kinds of differences in susceptibility among the microorganisms against antimicrobial substances in plant extracts may be explained by the differences in cell wall composition and/or inheritance genes on plasmids that can be easily be transferred among bacterial strains. Results of the present study suggested that the extracts from *C. nummularia* can be exploited as a potential source of natural agents (antioxidant, antibacterial, antimutagenic, and enzyme inhibitors) for the management of oxidative stress and global health problems. The data provides strong scientific evidence for traditional uses of *C. nummularia*. Finally, *in vivo* investigations are needed to test the biological effects of *C. nummularia* on human health.
